# From asymmetry to algorithm: AI-driven innovations in hemifacial microsomia reconstruction

**DOI:** 10.1097/MS9.0000000000004907

**Published:** 2026-04-13

**Authors:** Hamna Hassan Siddiqui, Syeda Aliza Aamir, Muhammad Talha, Mahnoor Fatima, Ubaid Ur Rehman

**Affiliations:** a Department of Medicine, Sindh Medical College, Karachi, Pakistan; b Department of Medicine, Liaquat University of Medical Health and Sciences, Jamshoro, Pakistan; c Department of Medicine, King Edward Medical University, Lahore, Pakistan; d Department of Medicine, Shaheed Ziaur Rahman Medical College, Rajshahi Medical University, Bogura, Bangladesh

*To the Editor*,

Hemifacial microsomia (HFM) is a congenital defect in the craniofacial area in which the facial structures that originate from the first and second pharyngeal arches develop unevenly. It stands second among all congenital craniofacial conditions, with a prevalence rate of about 1 in 5600 live births in the USA. The cause of the condition is said to be mainly due to the disruption of blood supply during the development of the embryo, especially concerning the stapedial artery; however, environmental and maternal risk factors have also been considered. HFM is diagnosed by the presence of facial asymmetry, loss of the mandible or external ear on one side, and changes in the carotid canal on the affected side. Patients usually undergo several reconstructive surgeries needing to repair nerves to attain better symmetry and function of the face. Though the death rate is very low, the long-term functional impairment is considerable; hence, the advanced therapeutic strategies are still needed[1].

Convolutional neural networks, algorithms that are efficient for image pattern recognition, have made very important contributions to craniofacial surgery by allowing whole processes from feature extraction to classification in one model. AI-connected diagnostic systems are capable of identifying craniofacial bone fractures in various locations, such as extremities, spine, and hip, with a mean diagnostic accuracy of 90.08%. Moreover, incremental generative adversarial networks have been used for converting computed tomography scans into magnetic resonance images, thus making the imaging process more efficient[2]. AI has also been shown to be effective in minimizing the workload of clinicians through the automation of sagittal X-ray parameter measurement, which is accompanied by high accuracy and time savings[3].

AI was primarily responsible for bringing about new innovations, the application of which, in turn, has created new methods of nose, chin, and tooth location identification, resulting in simplified device traceability, administrative cost-cutting, and overall clinical accuracy. Besides, AI-powered postoperative patient care monitoring systems that apply skin color changes and pulse-wave analysis have been credited with detecting wound healing complications at an early stage[4]. In neurorehabilitation, the AI-powered system for electrostimulation linked to the auricle has been devised to assist the facial muscles of patients with peripheral facial palsy. It has been able to elicit eye blinking, eye closure, and smiling on the affected side, besides reaching a macro F1-score of ρ = 0.570 that proves the performance statistically significant[5].

In spite of these innovations, there are still a number of shortcomings that prevent the large-scale use of AI-based neurotechnologies in craniofacial surgery. The majority of AI systems still need continuous human oversight and specific training in machine learning, which can counterintuitively add to the clinical workload rather than lessen it. Furthermore, the intricate and non-transparent nature of many AI algorithms is a barrier to human understanding and makes it harder for doctors to place complete trust in or accept systems whose routes of reasoning are still quite obscure. The absence of explainability is particularly alarming in medical practice since the decisions made by the algorithm have a direct impact on patient outcomes[2].

The union of artificial intelligence, neurotechnology, and regenerative craniofacial surgery indicates an area that is rapidly changing and not well covered by research yet. Integration of AI-assisted electrostimulation with the newly emerging nanomedical techniques has the potential to restore facial functions in cases of HFM. Continuous interdisciplinary teamwork among surgeons, data scientists, and bioengineers will be the key to converting algorithmic creativity into clinical practice. Besides, research on the necessary conditions of AI systems that are conversable and ethically sound will have great importance in the areas of patient safety, clinician trust, and regulatory acceptance. As technology increasingly becomes more precise, the scenario in facial reconstruction may go from being only anatomical correction to including total functional rehabilitation and biological regeneration.

This letter to the editor adheres to the Transparency in the Reporting of Artificial Intelligence in Research (TITAN) guidelines[6].

## Data Availability

Not applicable to this study.
